# An editor for pathway drawing and data visualization in the Biopathways Workbench

**DOI:** 10.1186/1752-0509-3-99

**Published:** 2009-10-02

**Authors:** Robert W Byrnes, Dawn Cotter, Andreia Maer, Joshua Li, David Nadeau, Shankar Subramaniam

**Affiliations:** 1 San Diego Supercomputer Center, University of California - San Diego, 9500 Gilman Drive, La Jolla, CA 92093-0505, USA; 2 Departments of Bioengineering, Chemistry and Biochemistry, University of California-San Diego, 9500 Gilman Drive, La Jolla, CA 92093-0612, USA; 3 ICx Biosystems, 505 Coast Blvd. South, Ste. 309, La Jolla, CA 92037, USA; 4 9500 Gilman Drive, MC-0612, San Diego, CA 92093-0612, USA

## Abstract

**Background:**

Pathway models serve as the basis for much of systems biology. They are often built using programs designed for the purpose. Constructing new models generally requires simultaneous access to experimental data of diverse types, to databases of well-characterized biological compounds and molecular intermediates, and to reference model pathways. However, few if any software applications provide all such capabilities within a single user interface.

**Results:**

The Pathway Editor is a program written in the Java programming language that allows de-novo pathway creation and downloading of LIPID MAPS (Lipid Metabolites and Pathways Strategy) and KEGG lipid metabolic pathways, and of measured time-dependent changes to lipid components of metabolism. Accessed through Java Web Start, the program downloads pathways from the LIPID MAPS Pathway database (Pathway) as well as from the LIPID MAPS web server . Data arises from metabolomic (lipidomic), microarray, and protein array experiments performed by the LIPID MAPS consortium of laboratories and is arranged by experiment. Facility is provided to create, connect, and annotate nodes and processes on a drawing panel with reference to database objects and time course data. Node and interaction layout as well as data display may be configured in pathway diagrams as desired. Users may extend diagrams, and may also read and write data and non-lipidomic KEGG pathways to and from files. Pathway diagrams in XML format, containing database identifiers referencing specific compounds and experiments, can be saved to a local file for subsequent use. The program is built upon a library of classes, referred to as the Biopathways Workbench, that convert between different file formats and database objects. An example of this feature is provided in the form of read/construct/write access to models in SBML (Systems Biology Markup Language) contained in the local file system.

**Conclusion:**

Inclusion of access to multiple experimental data types and of pathway diagrams within a single interface, automatic updating through connectivity to an online database, and a focus on annotation, including reference to standardized lipid nomenclature as well as common lipid names, supports the view that the Pathway Editor represents a significant, practicable contribution to current pathway modeling tools.

## Background

Pathways may be broadly described as models that characterize movement of material through a network of molecular species and processing steps. They serve as the basis upon which much of the new field of systems biology must build. Many tools have become available over the last 10 years for enabling biological pathway construction [1-7]. Their construction has been stimulated by the growth in information resulting from adoption of new laboratory tools accompanying high-throughput data acquisition, such as mass spectroscopy [8,9]. The process of constructing pathways requires ready access to information in the form of experimental data of a quantitative nature.

In general, the field of metabolomics involves inducing perturbations to the ongoing state of living systems and subsequently monitoring changes to compounds at specific time points. The interactions among components of a pathway are then inferred by a variety of techniques, including metabolite fingerprinting and profiling, and by comparison between organisms that have been genetically perturbed or subjected to altered nutritional states [8,9]. To serve as a resource for designing pathways, we have constructed an on-line database resource, named Pathway, from which metabolomic data may be obtained. In addition to KEGG reference lipid metabolic pathways, the Pathway database contains lipidomic, microarray, and protein array data acquired by member laboratories of the LIPID MAPS (Lipid Metabolites and Pathways Strategy) consortium. The shared goal of these member laboratories is to characterize the lipid metabolite component of cellular metabolism ("lipidomics") in the mouse and in humans [9,10]. Pathway also contains annotations derived from KEGG LIGAND COMPOUND and KEGG LIGAND ENZYME [11].

The Pathway Editor is the initial application of the Biopathways Workbench project. The Biopathways Workbench is an on-going effort of LIPID MAPS to build a programming toolkit, applications, and a database for creating, managing, visualizing, and editing signaling and metabolic pathways. The Biopathways Workbench makes extensive use of the connectivity that is possible between data sources provided by the internet and the local file system of a user. The toolkit includes capability for read-only access to Pathway and other databases, and for reading and writing different file formats.

Brief descriptions of the Biopathways Workbench and the Pathway Editor during early development have been presented previously [10].

## Implementation

### The Pathway database

Pathway is implemented as a relational database, using Oracle9i Enterprise Edition Release 9.2.0.2.0, running on a Sun Fire 880 server and maintained at the San Diego Supercomputer Center, University of California - San Diego by the LIPID MAPS consortium's Bioinformatics core. At the time of writing, the database contains LIPID MAPS experimental data acquired from treatments of RAW 264.7 cells, a cell line derived from macrophage tumor cells [12]. The cells were treated with Kdo_2_-lipid A, a purified component of bacterial lipopolysaccharide (LPS) which may be responsible for much, if not most, of the pro-inflammatory response to LPS. LPS mimics processes of a similar nature occurring in many disease states and conditions, including inflammation, atherosclerosis, and altered growth [13]. Lipids from treated cells were extracted and quantified by member laboratories of LIPID MAPS. Pathway also includes microarray and protein array time course measurements [14]. Descriptions of experimental protocols, as well as experimental data in other formats, may be viewed and downloaded from [15]. All of the information contained with the Pathway database is found elsewhere and is collected in a single database for convenience. Pathway is accessed by means of the Pathway Editor program and is not intended to be accessed through other means.

### The Pathway Editor program

The Pathway Editor is written in the Java programming language and is downloaded as a Java Web Start application from the LIPID MAPS website [16]. Web Start is a feature of the Java Runtime Environment (JRE) distributed by Sun Microsystems (Sun Microsystems Inc., Santa Clara, CA, USA). The current download requires prior or co-installation of JRE v. 1.5.0 or later version. Web Start checks the originating web site for updates automatically, and subsequently accesses them, with a minimum of interaction required of the user. Prior installation of libsbml [17,18] is required on the part of the user. Depending upon the operating system, the downloaded Pathway Editor application includes SBML (Systems Biology Markup Language) v 3.2.0 dynamic link libraries [18] for use on Windows desktops (dlls) or Java native interface libraries for use on Mac OS X desktops (jnilibs), as well as SBML Java files and other open-source libraries in Java. These open-source libraries are described elsewhere in this paper. The program is digitally signed to increase acceptability by security-conscious internet users.

The Pathway Editor appears as native look and feel applications on Microsoft Windows and Apple Macintosh operating systems. Modern graphics accelerator hardware (such as is installed in current Microsoft Vista-ready and Macintosh desktop computers) is required. A link to a tutorial on use of the Pathway Editor may be found on the Pathway Editor download page [16].

### Design of the Pathway Editor

The design of Pathway Editor follows the Model/View/Controller (MVC) design pattern [19]. The Model is composed of Biopathways Workbench classes that access files and databases. The DocumentWindow class represents the Controller, with which the user interacts to select information and to set view preferences. The View includes the VisPathwayPanel class, which draws and presents pathway diagrams to the user. In typical usage, a Pathway object containing layout information and time course data is created in the Biopathways Workbench and conveyed to the DocumentWindow and the VisPathwayPanel, and returned to the Workbench for updating or storage in the file system. Alternatively, a pathway may be constructed by combined use of the DocumentWindow and VisPathwayPanel and sent to the Workbench for transmittal to the user's file system.

The Pathway Editor constructs pathway objects using a standardized object vocabulary that includes small molecules, proteins, nucleic acids, experiments, and time course datasets. Code representations of nodes and interactions within a pathway are called participants, and contain information that is used for drawing on the VisPathwayPanel (for example, label text, coordinates, shape, color, connectivities, and so on). Node participants may also contain experiment data.

Complete pathway diagrams may be saved as .path files in XML format for future viewing in the Editor, or as image files of the drawing surface in various formats.

Additional implementation details are described in the Results section.

## Results

### Pathway database

The focus of data management in Pathway is the experiment, identified by an experiment ID (identifier) and its associated metadata (including LIPID MAPS laboratory, the broad category of lipid studied by the lab, biological system, type of experiment, and experiment date). An experiment contains all of the measurements and compounds or gene symbols for treatments performed by a particular laboratory on a particular date in the form of sets of time course data, or datasets. A dataset consists of a string representing a treatment type and a series of time points (0, 0.5, 1, etc). The dataset contains one or more measurements at each time point. Time units are considered as part of the enveloping experiment, while measurement units are contained within the dataset.

The Pathway database also contains references to small molecules as identified by LIPID MAPS structure database identifiers [20,21] and to KEGG small molecule compound IDs, to KEGG protein compound IDs, and to gene symbols referenced by Entrez Gene [22]. Microarray and protein array measurements within the database are associated with both a protein compound ID and a gene symbol. That is, the Pathway Editor treats array data as belonging to proteins. Nucleic acid annotations are not currently found in Pathway.

When experiments are loaded into the Pathway database, each dataset becomes associated with a corresponding small molecule ID, protein ID, or combined protein identifier plus gene symbol identifier. The dataset of a compound thus becomes linked to the characteristics of the compound, such as small molecule category, molecular weight, synonyms, comments, and the external database identifiers described in the preceding paragraph. Further details on experiments and datasets may be found in the on-line tutorial [16].

The Pathway database contains KEGG reference metabolic pathways for lipid metabolism. The components of the pathways are parsed from KEGG XML files originally written in the KEGG Markup Language (KGML), and include pathway name and compound or protein name assigned to pathway nodes, node connectivity and stoichiometry, their respective map coordinates, and KEGG database IDs for KEGG LIGAND COMPOUND and KEGG COMPOUND ENZYME [11]. If the KEGG compound has been identified as equivalent to a structure contained in the LIPID MAPS structure database [20], the Pathway database characterization of the node in the KEGG pathway also contains the Pathway small molecule or protein ID described in the previous paragraph, allowing access to all annotations available for that molecular entity.

### Visualization and data management

**Figure 1:**
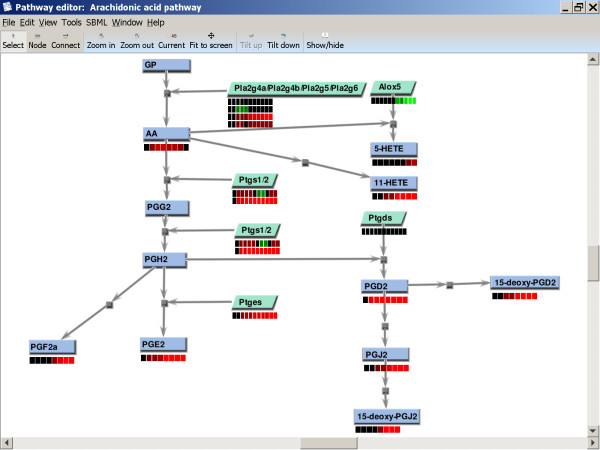
**Representative LIPID MAPS pathway in Pathway Editor**. The average of 3 lipidomic experiments and one microarray experiment is shown in the default heatmap display format. Shades of red indicate an increase in the ratio of metabolite or gene expression in treated cells with respect to untreated controls measured at the same time point; shades of green represent a reduction in the ratio, or repression of expression. A figure illustrating the alternative line chart display format is shown in [9]. Small molecules are shown as blue rectangular nodes, while protein nodes are shown as green parallelograms (both default shapes for these node types). The default settings for opening a .path file (see text) also feature automated fit to the viewing panel and adjustment of the zoom to the setting contained in the file. View and pathway preferences for any or all nodes and interactions are configurable. The Arachidonic acid metabolism pathway [16] is shown.

A view of the Pathway Editor showing a sample LIPID MAPS pathway (the Arachidonic acid pathway) is shown in Figure 1. A drawing area in the lower portion of the window frame comprises most of the display. Drawing is performed using JOGL (Java Open Graphics Language) [23] to simulate a three dimensional display. The display of time course data for every node, for all nodes of a particular type, or for individual nodes may be configured as either heatmap or line chart. Heatmaps permit more efficient usage of screen space than line charts. Above the drawing area is a toolbar containing buttons that may be clicked to provide many commonly-used functions (i.e., node select, node create, and node connect modes, zoom in, zoom out, a zoom display area, fit to screen, tilt view plane up, tilt view plane down, and show/hide data display). The user may move scroll horizontally or vertically across a plane in 3D space perpendicular to the user's line of sight using scroll bars. Above the toolbar, and topmost in the frame, is a menu containing common program interface menus, as well as specialized submenus.

View preferences, such as node/interaction color, shape, font size, and others may be configured by a preference assignment dialog accessed from the popup menu, or by global preference assignment dialogs accessed on the View menu.

The program also uses the JFreeChart code package [24] to generate high-quality charts in separate frames for close inspection of data.

### Downloading pathways

**Figure 2:**
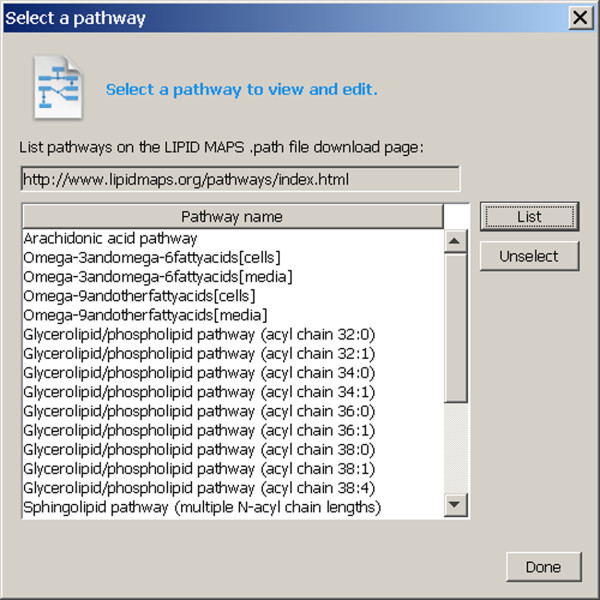
**LIPID MAPS pathway download dialog**. The LIPID MAPS pathway download dialog is obtained by selecting "Open LIPID MAPS pathway database..." from the File menu. "LIPID MAPS pathway database" is used in a general sense to indicate a page on the LIPID MAPS website, as a storage location for pathway files. The act of clicking on any row of the table directs the Pathway Editor to download and display the pathway.

The user may initiate a session with the Pathway Editor in a number of ways. Perhaps the most powerful mode may be entered by downloading a pathway. A pathway file in the Biopathways Workbench format (ending with the extension .path) may be downloaded from the LIPID MAPS website server [16] by means of a dialog presented via the File menu (Figure 2). .path files are built using the Pathway Editor and contain a listing of the nodes and processes of a pathway, along with their geometrical layout and Pathway database identifiers of compounds referenced by each node. The files may also contain experiment IDs, if a pathway designer desires. The presence of an experiment ID directs the Editor to download all datasets for the experiment from the Pathway database. If the experiment happens to be a microarray or protein array experiment, only the time course data for specific gene symbols that are referenced in the file are downloaded, because of the large number of datasets in these kinds of experiments. The Editor traverses each participant in the pathway, and determines whether a Pathway database identifier for the participant compound is present in the list of compounds in each experiment. If data is found, datasets are copied from the experiment to the participant object that is represented by the node. The data is displayed in the drawing area along with the pathway (Figure 2). The default display format is heat map; this may be changed to line chart as desired for each node, or for all nodes of any type. .path files on the user's machine may also be accessed.

**Figure 3:**
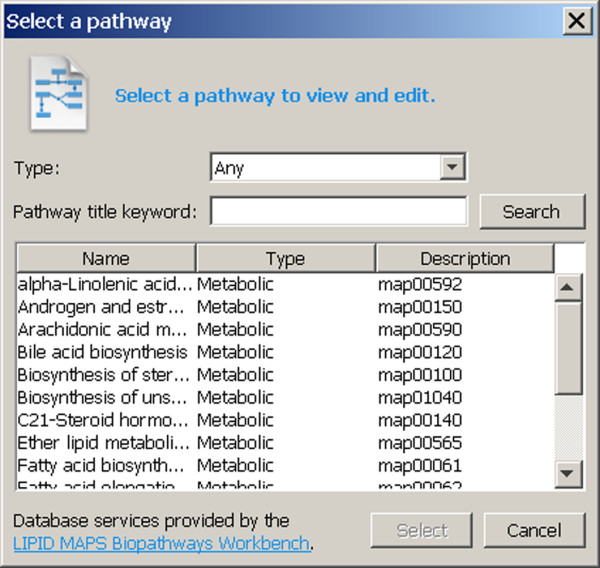
**KEGG pathway download dialog**. The KEGG pathway download dialog is obtained by selecting "Open KEGG pathway database..." from the File menu. The user may press the Search button to list all KEGG pathways in the Pathway database. The user can restrict the search by first selecting the type of pathway and entering a keyword contained in a title, if desired, prior to pressing the Search button. Clicking on any row of the table directs the Pathway Editor to download and display the pathway.

KEGG pathway files may be downloaded directly from Pathway, again using a dialog available through the File menu (Figure 3). If the database representation of the KEGG pathway is cross-referenced to Pathway compound identifiers, experiment data is assigned by the Pathway Editor in a manner similar to LIPID MAPS .path files. However, references to experiments are not contained within KEGG pathway files, and experiment data must be accessed in separate steps (see the following discussion).

Nodes on the drawing surface of the Pathway Editor are of four types, following KEGG terminology: small molecule, protein, nucleic acid, or unknown (the default type). Each node (i.e., its internal representation as a participant) may contain a referenced compound ID. Protein nodes may also contain a list of one or more gene symbols. Processes, or interactions that connect nodes, are of type metabolic, signaling, or unknown (the default type). These compound IDs, symbols, and types may be assigned using dialogs accessible via mouse buttons and top menu items.

The user assigns compounds (i.e., small molecules or proteins) to nodes by accessing a database search dialog and searching the Pathway database. The search may utilize compound and synonym names or fragments of names. Once assigned, the Pathway database compound ID and compound metadata becomes associated with the node. Compound metadata is updated automatically from the database whenever a .path file is downloaded or opened.

Nodes are created by combined use of toolbar buttons and mouse actions. To create a node in any part of the drawing area, the *Node create *button in the toolbar is pressed and the drawing area is clicked. To connect nodes, the *Node connect *button is pressed, a node is selected, and, keeping the mouse button pressed, the mouse is moved to a second node, and the mouse is released. The resulting interaction, or process, may be assigned information using the mouse and pop-up dialogs, in a manner similar to nodes.

**Figure 4:**
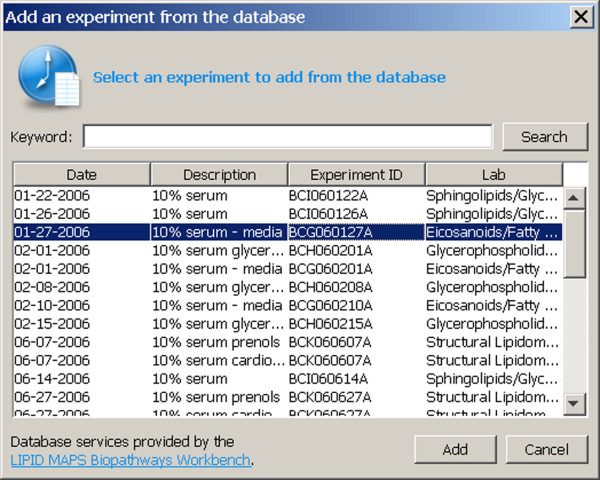
**Metabolic experiment download dialog**. The metabolic experiment download dialog is obtained by selecting "Manage time course experiments..." from the Tools menu. The user may press the Search button to list all experiments in the Pathway database. The user can restrict the search by first entering a keyword contained in the tile, if desired, prior to pressing the Search button. Clicking on any row of the table and then pressing "Add" directs the Pathway Editor to download and display the data for the experiment. The "Cancel" button cancels an ongoing download.

Within the Pathway Editor, experiments are managed independently of pathway components. When an experiment is presented to the Pathway Editor, the Pathway Editor automatically associates datasets with nodes containing the compound ID or gene symbol for the dataset. Experiments may be downloaded from the Pathway database separately from pathways (Figure 4), or may be accessed from the local file system. In the latter instance, the files may be in LIPID MAPS data file format (containing one or more experiments that have been previously downloaded in the Pathway Editor and saved by the user), or the files may be in comma-separated value (CSV) format and constructed by the user by way of another program, such as a spreadsheet application [16]. The CSV format does not contain database identifiers (experiment or compound). Such numerical identifiers are created and assigned by the Pathway Editor as necessary. In the case of compounds (node type protein or small molecule), identifiers are assigned on the basis of 1:1 correspondence between names in the CSV file and node labels in the pathway. Microarray or protein array data in CSV files is identified by a node type designation of protein, in combination with a gene symbol.

### Assigning a compound to a node

During the process of constructing a pathway, a user constructs a node of the desired type, and assigns a compound to a node by searching Pathway for the name of a compound or gene symbol, using a compound information dialog for the node type. Once found and selected, the Pathway database ID for the compound and/or gene symbol then becomes incorporated into the participant object that is contained in the local pathway. If experiment data is loaded in the program, the program then automatically traverses the experiment data contained within the internal pathway object and assigns datasets containing the same database compound ID or gene symbol to the participant contained within the node for display.

**Figure 5:**
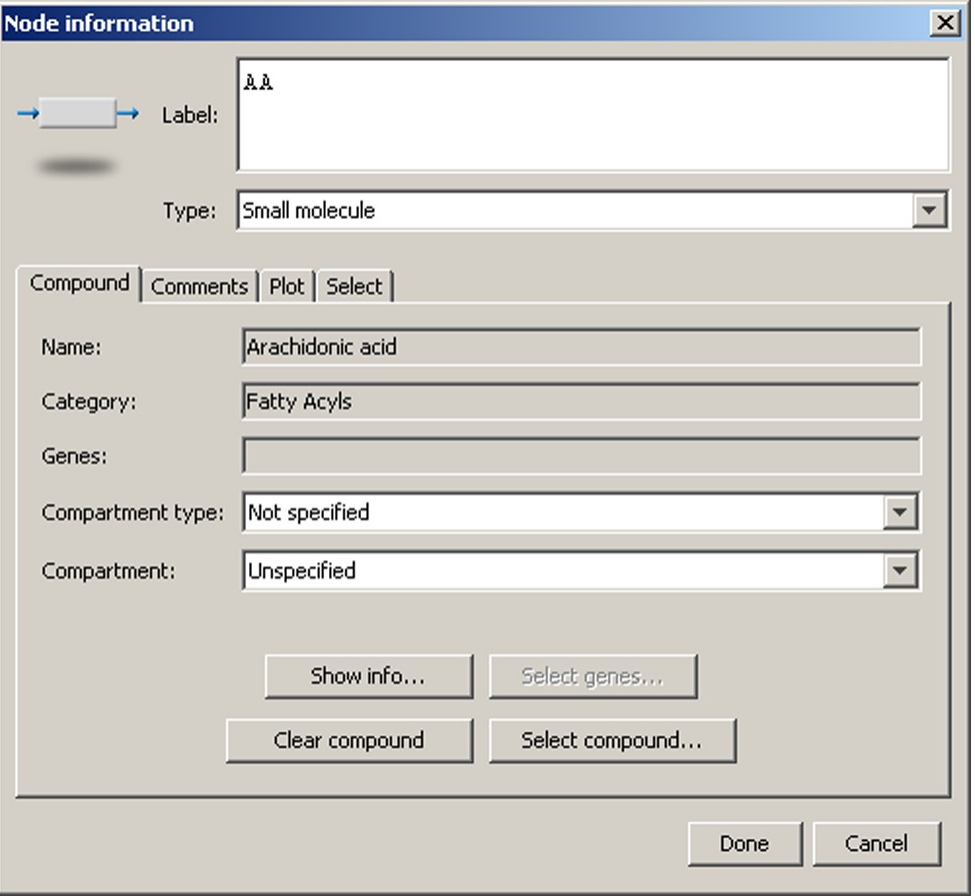
**Node information dialog**. The node information dialog is obtained by selecting a node, right-clicking and selecting "Show info..." from the pop-up menu. The dialog is also reachable via the Tools menu. The dialog is used to enter a label for the node, shown on the drawing panel, and to select the type of molecule, which in turn changes the display properties of the node. The user may also enter a compartment type (Not specified, Cellular, or Intracellular) and a compartment (restricted to Gene Ontology cellular components [33,34]). The "Select genes..." and "Select compound..." buttons bring up dialogs allowing search and selection of gene symbols and compounds in the Pathway database for assignment to the node. Other tabs allow the user to enter comments into the pathway for the node, to examine a heat map or line chart of data and numeric values, and to set the visibility of the data on the main drawing panel for the compound or gene symbols. The node information dialog for Arachidonic acid is shown.

A user accepts placement of the compound in the node on the basis of whether the measured data that is presented meets with expectations according to domain knowledge, including early or late responsiveness to a stimulus, and the magnitude of the response. Measured absolute values and ratios may be inspected, and the presentation changed, by right-clicking on an interaction glyph and selecting from a pop-up menu with the mouse, or alternatively, by accessing menu items on the Tools menu for the window. For microarray experiments, the presented data includes statistical p-values of the significance of biological replicates (experiments performed on different dates), technical replicates (multiple within-experiment replicates), or treatment significance (i.e., Kdo_2_-lipid A vs. control treatments) on the Select tab of the Node information dialog (Figure 5).

A menu item in the View menu enables animated bar charts for visualization of time-dependent changes for each node in the display. This allows dynamic comparisons of the magnitude and direction (up or down) of changes to compounds in the system under study.

The user is further aided in the process of compound assignment by having the ability to change the visibility of entire experiments, of datasets for individual compounds, and of datasets for individual gene symbols in the display, as desired. The user may choose to reject assignment of a compound to a node because of poor reproducibility of measurement, or may reject an individual experiment because of inconsistency within the experimental data, thus accepting the compound.

**Figure 6:**
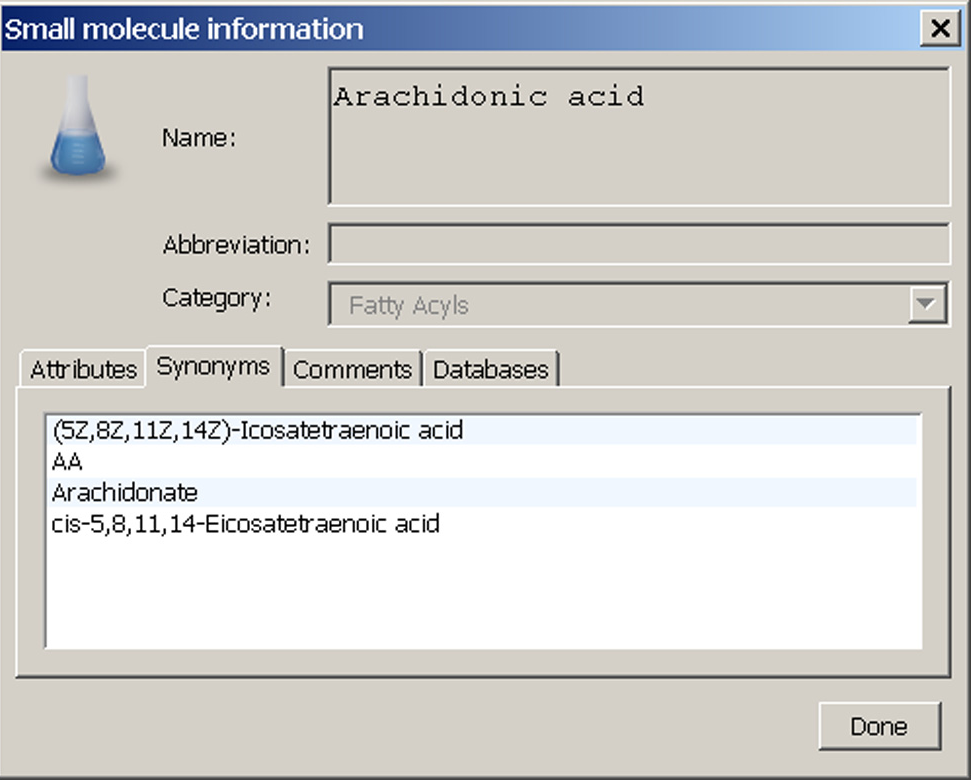
**Small molecule compound information dialog**. The small molecule compound information dialog is obtained by pressing the "Show info..." button on the node information dialog for a small molecule node (see Figure 5). The dialog displays information on the compound that is contained within the Pathway database and is not editable by the user. Analogous dialogs exist for protein and for nucleic acid nodes. The small molecule compound information dialog for Arachidonic acid is shown.

Further information on the compound may be obtained by double-clicking a table row containing an identifier in the databases tab of the compound's information dialog, as in Figure 6. The action causes the user's preferred browser program to open a web page presenting database information from LIPID MAPS, KEGG, or Entrez Gene for that identifier.

### SBML

The Pathway Editor reads and writes Systems Biology Markup Language (SBML) models through level 2, version 3 [17,18,25]. SBML species and reactions can be assigned to pathway nodes and interactions. When saving an SBML model, the LIPID MAPS pathway layout is contained within the model as an annotation. This layout is automatically utilized when the file is subsequently read. If a pathway layout is not found, the user may select an automated layout feature patterned after that for JDesigner [26], or the user may manually select nodes from a list and paste them on the drawing surface one at a time. Interactions are automatically drawn, utilizing lists of SBML species and reactions contained within the model.

The SBML plugin in Pathway Editor is designed to be fully compatible with the SBML specification [18]. Mathematical expressions relating SBML species, parameters, and compartments can be written and viewed. Of particular interest is the ability to enter and read MIRIAM annotations (Minimal Information Required for the Annotation of Mathematical Models) and SBO (Systems Biology Ontology) references [27-29] in user-selectable table and list format. Upon double-clicking on a row in the table, the user is presented with the database web page for the compound in the user's browser, in a manner similar to database references presented for .path files, above.

## Discussion

Tools for constructing and editing pathways require drawing on readily available information of various types. Features of the Pathway Editor that assist in providing this information are: First, it provides simultaneous access to time course (metabolomic) and microarray measurements, as well as protein array data. Intermediates in pathways are identified with reference to compound names as defined by an ongoing effort to standardize lipid nomenclature (LIPID MAPS) [20,21] as well as to common names of lipids, and to gene symbols. Compounds that are present in KEGG databases [11] are also identified. Performance of additional steps to acquire data is not necessary, as may be the case with other pathway analysis tools that require access to diverse sources. Second, it provides automated access to annotations and to experimental data, incorporating automated updating. Third, users may create and edit new and existing pathways by adding and deleting nodes and processes. Users may incorporate their own metabolites and measurements during pathway construction. Lastly, access to new databases and input/output of new pathway model types is facilitated by a program structure that was designed with this in mind from the beginning. To our knowledge, this combination of features in a single program is unique to the Pathway Editor.

Of the several tools available, VANTED [6] may perhaps come closest to the functions of the Pathway Editor; however, its use of on-line databases is limited to KEGG, and experimental data must be loaded into the program from files. Display of heatmaps is not supported. Finally, the compatibility of VANTED with SBML specifications is incomplete (see ).

BiologicalNetworks [1] is a cross-database application that integrates disparate organisms and data types, but access to metabolomic data is not provided. PathwayExplorer [2] maps gene expression data to pathways, displaying the data in heatmap format, but does not allow for manipulation of the pathway, or creation of new pathways. Thus, the Pathway Editor represents a significant contribution to the list of current tools for pathway construction.

Small molecules in the Pathway database are annotated with reference to LIPID MAPS lipid identifiers and KEGG small molecule IDs. In some cases, the precise stereochemistry of a measured lipid may not be known because of ambiguity in structure assignment [30]. The time course data for such compounds is nevertheless included in Pathway, with this proviso, to serve as placeholders until more precise determination of structures is made.

For constructing pathways de novo, the Pathway Editor currently depends upon user interaction. The process of pathway construction relies heavily on domain knowledge. However, the focus of the Pathway Editor is on access to data, its graphical representation, and annotation. It is hoped that its appealing visual design, richness of features, simplicity of default preferences, and responsiveness, as well as similarity of the descriptive terminology used by the program (i.e., experiment, treatment, etc.) with laboratory methodology, will support facile use of the Pathway Editor by experimenters.

### Future enhancements to the Pathway database

Pathway is enhanced with additional experimental data as it is acquired and validated. Currently, LIPID MAPS laboratories are using several types of primary macrophages from mice as target systems. RAW 264.7 subcellular fractions are also being studied. Lastly, treatments with different agents, including an inhibitor of cholesterol metabolism, compactin, in conjunction with Kdo_2_-lipid A, as well as models for minimally modified LDL (low density lipoprotein) [31] are under way or planned.

### Enhancements to the Biopathways Workbench and the Pathway Editor

The Biopathways Workbench component of the Pathway Editor interfaces with the Pathway database and with the local file system. It converts textual and numeric information into data structures that can be transferred to the rest of the application. These data structures represent concrete biological entities and comprise the core of a vocabulary which can be extended as necessary as other databases are accessed and new analysis features are incorporated in the Pathway Editor and other applications.

A number of extensions to the Pathway Editor, making use of Biopathways Workbench toolkit classes, are planned. Support for additional pathway model file formats, such as BioPAX [32] will be available in the near future. Provision of access to other experiment databases is anticipated. We are also working on providing a form of the program that does not require a connection to the internet. The ability to perform statistical analyses that are important in the systems biology field will be added. Lastly, more extensive use of 3D graphics, particularly in the area of compartmentalized localization of processes and interchange of compounds between compartments, is also anticipated.

Investigators may contact the authors to discuss requests to add on-line access to their data and compound databases in the Pathway Editor.

## Conclusion

The Pathway Editor provides a user interface that features multiple functions for rapid pathway construction, including access to multiple pathway diagrams and experimental data sets; a focus on annotation, including references to standardized lipid nomenclature as well as common lipid names; and automatic updating of experimental data and annotations through connectivity to an online database. This rich feature set is not available in any existing pathway drawing tool.

## Availability and Requirements

Project name: Biopathways Workbench

Project home page: 

Operating systems: Windows, Apple Mac

Programming language: Java

Other requirements: Java 1.5 or higher; installation of libsbml

License: Freely available

Restrictions to use by non-academics: None

## Authors' contributions

RWB wrote Pathway Editor code, proposed features of the Pathway Editor, designed the Pathway database, and drafted the manuscript; DC designed the Pathway database and critiqued the manuscript; AM proposed features of the Pathway Editor and designed the Pathway database; JL designed the Pathway database; DN wrote Pathway Editor code, proposed features of the Pathway Editor, and designed the Pathway database; SS proposed features of the Pathway Editor and critiqued the manuscript. All authors read and approved the final manuscript.
